# Recanalization of the Paraumbilical Vein Mimicking an Umbilical Hernia: A Case Report

**DOI:** 10.7759/cureus.118472

**Published:** 2026-10-23

**Authors:** Giovana Arcuri Cavalcanti, Bruna Alves de Arruda Ferreira, Ana Sofia Faria Pereira, Olival C da Fonseca Neto

**Affiliations:** 1 Medical School, Universidade de Pernambuco, Recife, BRA; 2 Internal Medicine, Universidade de Pernambuco, Recife, BRA; 3 Internal Medicine, Hospital Universitário Oswaldo Cruz, Recife, BRA; 4 Surgical Gastroenterology, Hospital Universitário Oswaldo Cruz, Recife, BRA

**Keywords:** caput medusae, chronic liver conditions, colored flow doppler ultrasound, ultrasound, umbilical hernia repair

## Abstract

Portal hypertension associated with chronic liver disease may lead to recanalization of the paraumbilical vein and the development of portosystemic collateral vessels, classically manifesting as caput medusae. Although this finding is uncommon in contemporary clinical practice, dilated paraumbilical collaterals may present as an umbilical protrusion and mimic an umbilical hernia. Recognition of this condition is crucial because inadvertent surgical injury to these vessels may result in severe, potentially life-threatening hemorrhage. We report a case of a 77-year-old male with compensated hepatitis C virus-related cirrhosis (Child-Pugh class A; Model for End-Stage Liver Disease (MELD) score 10) who presented with an asymptomatic, reducible 2 cm umbilical protrusion initially considered an umbilical hernia. Doppler ultrasonography of the abdominal wall was performed and demonstrated a patent, recanalized paraumbilical vein communicating with the left portal vein branch and extending into the superficial abdominal wall, with drainage toward the epigastric and femoral venous systems. These findings were consistent with extensive portosystemic collateral circulation, representing caput medusae and accounting for the apparent umbilical protrusion. Recognition of the vascular nature of the lesion prevented an unnecessary surgical intervention and its associated hemorrhagic risk. This case emphasizes the importance of considering portosystemic collateral vessels in the differential diagnosis of umbilical protrusions in patients with chronic liver disease. Differential diagnoses include true umbilical hernia, isolated abdominal wall varices, venous malformations, and metastatic umbilical nodules such as Sister Mary Joseph's nodule. Doppler ultrasonography is a readily accessible, noninvasive method for characterizing the lesion, demonstrating vascular flow, identifying communication with the portal venous system, and defining the extent of collateral circulation. Therefore, vascular imaging should be considered before surgical repair of an apparent umbilical hernia in patients with cirrhosis or other conditions associated with portal hypertension. Caput medusae may present as an umbilical protrusion mimicking a hernia, and careful clinical assessment combined with Doppler ultrasonography is essential for accurate diagnosis and prevention of potentially catastrophic surgical hemorrhage.

## Introduction

Chronic liver disease is responsible for approximately 10,000 deaths annually, constituting a major public health problem in Brazil [1]. One of the most severe and clinically significant consequences of this condition is portal hypertension, characterized by increased pressure within the portal venous system, often resulting from elevated intrahepatic vascular resistance secondary to hepatic fibrosis [2,3].

Caput medusae is a clinical manifestation associated with portal hypertension, characterized by dilated, tortuous superficial veins radiating from the umbilicus and resembling the mythological figure of Medusa [2]. Its development reflects the formation of portosystemic collateral circulation in response to increased portal pressure. The umbilical vein normally obliterates after birth and becomes the ligamentum teres hepatis. In portal hypertension, the paraumbilical venous channels may become prominent or recanalized, allowing blood to flow from the portal venous system into superficial veins of the anterior abdominal wall [2,3]. When this collateral circulation produces the characteristic visible venous pattern, the clinical sign is termed caput medusae.

Recanalization of the paraumbilical vein is also associated with the Cruveilhier-Baumgarten spectrum, which classically involves portal hypertension, paraumbilical venous collateralization, and an abdominal venous hum. However, the presence of paraumbilical vein recanalization alone does not establish this syndrome. These vascular abnormalities can be evaluated using imaging modalities, particularly Doppler ultrasonography, computed tomography, and magnetic resonance imaging [2,4,5].

Umbilical hernias are also common in patients with cirrhosis, particularly in the presence of ascites, increased intra-abdominal pressure, and weakening of the abdominal wall. Consequently, a periumbilical protrusion in a patient with chronic liver disease may represent an umbilical hernia, vascular collateralization, or potentially both. Distinguishing these conditions is clinically important because inadvertent injury to dilated portosystemic collaterals during surgery may cause severe hemorrhage [3,4]. Doppler ultrasonography is a valuable, noninvasive tool for evaluating periumbilical abnormalities because it can demonstrate venous flow within collateral vessels and help differentiate vascular lesions from abdominal wall hernias [5].

This report describes a patient with chronic liver disease who presented with an umbilical protrusion initially suspected to be an umbilical hernia but was found on Doppler ultrasonography to have a recanalized paraumbilical vein. The case highlights the importance of recognizing paraumbilical venous collateralization as a differential diagnosis of umbilical masses and using appropriate imaging to guide clinical assessment and avoid potentially hazardous interventions.

## Case presentation

A 77-year-old male with a medical history of systemic arterial hypertension and benign prostatic hyperplasia was referred to the hepatology outpatient clinic approximately two years ago following the identification of thrombocytopenia during routine preoperative laboratory testing for a urological procedure. During the etiological investigation, chronic hepatitis C virus (HCV) infection was diagnosed, confirmed by reactive anti-HCV serology and the detection of HCV-RNA. The patient was treated with sofosbuvir and velpatasvir, with dosages per institutional protocol, for 12 weeks; the course was completed without adverse events or clinical complications. Twelve weeks after the conclusion of treatment, he achieved undetectable HCV-RNA, characterizing a sustained virological response (SVR), and remained in asymptomatic outpatient follow-up. The patient continued taking losartan, doxazosin, and finasteride for his pre-existing comorbidities.

During a follow-up consultation, a painless, compressible umbilical protrusion measuring approximately 2 cm was identified on physical examination (Figure 1). The lesion was soft, with normal overlying skin coloration, and showed no apparent changes with coughing, straining, or standing. No venous hum was detected on auscultation.

**Figure 1:**
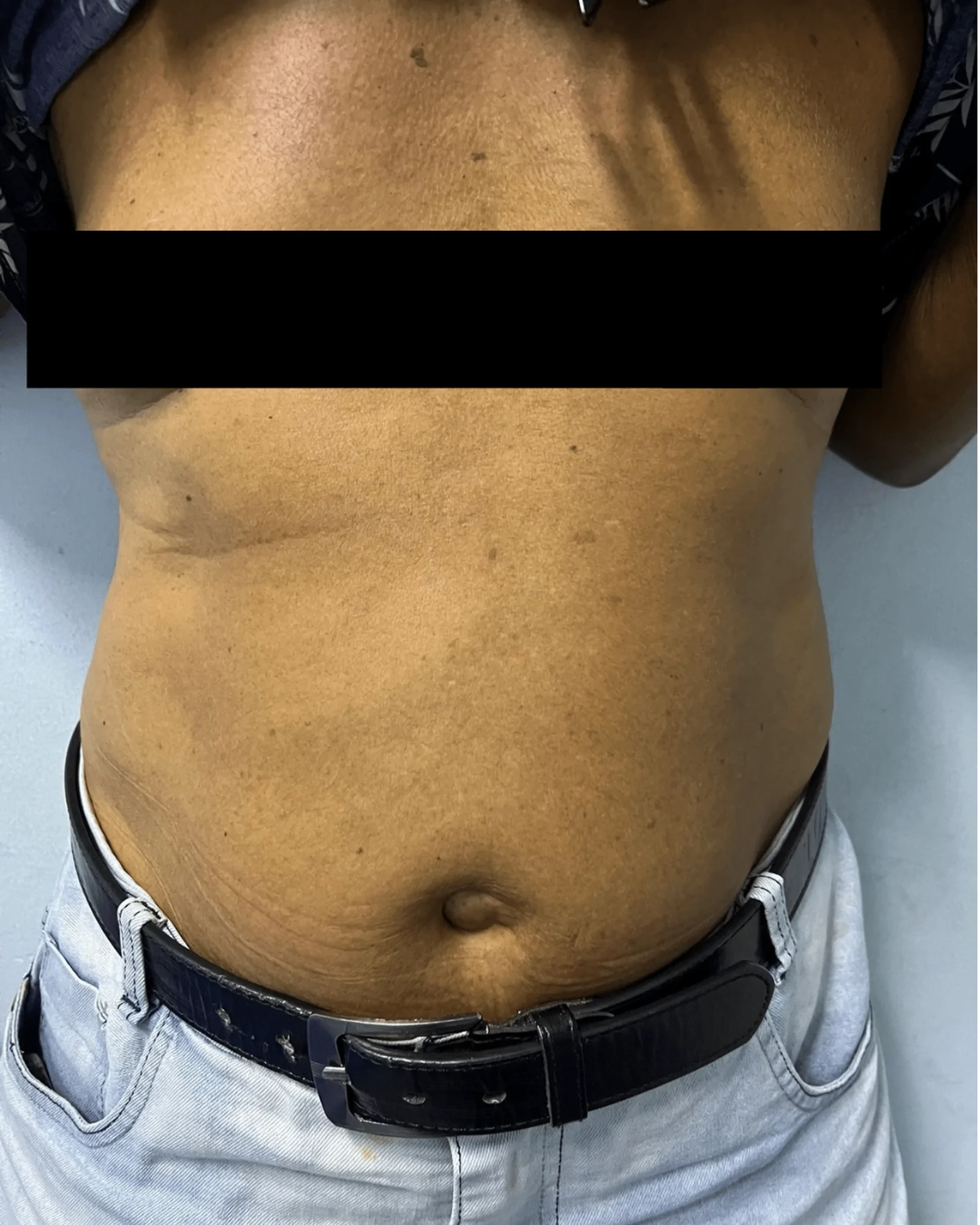
Anterior abdominal wall showing umbilical prominence.

At that time, the patient showed no clinical manifestations of hepatic decompensation. Laboratory results were as follows: aspartate aminotransferase (AST), 25.3 U/L (reference range (RR): 10-30 U/L); alanine aminotransferase (ALT), 39 U/L (RR: 10-50 U/L); total bilirubin, 1.8 mg/dL (RR: 0.1-1.1 mg/dL), with direct bilirubin of 0.7 mg/dL and indirect bilirubin of 1.1 mg/dL; platelet count, 92,000/µL (RR: 150,000-450,000/µL); international normalized ratio (INR), 1.35; creatinine, 0.7 mg/dL (RR: 0.7-1.4 mg/dL); albumin, 4.1 g/dL (RR: 3.4-4.8 g/dL); and serum sodium, 140 mmol/L (RR: 135-145 mmol/L). Laboratory results are presented in Table 1. Based on these findings, the patient was classified as Child-Pugh class A (5 points), with a Model for End-Stage Liver Disease 3.0 (MELD 3.0) score of 11, consistent with preserved hepatic function despite imaging findings indicative of portal hypertension (Appendices) [6,7].

**Table 1:** Laboratory results.

Laboratory	Patient value	Reference range
Aspartate aminotransferase (AST)	25.3 U/L	10–30 U/L
Alanine aminotransferase (ALT)	39 U/L	10–50 U/L
Total bilirubin	1.8 mg/dL	0.1–1.2 mg/dL
Direct bilirubin	0.7 mg/dL	0.0–0.3 mg/dL
Indirect bilirubin	1.1 mg/dL	0.2–0.9 mg/dL
Platelet count	92,000/µL	150,000–450,000/µL
International normalized ratio (INR)	1.35	0.8–1.2
Creatinine	0.7 mg/dL	0.7–1.4 mg/dL
Albumin	4.1 g/dL	3.4–4.8 g/dL
Sodium (Na)	140 mmol/L	135–145 mmol/L

The patient expressed interest in elective surgical correction; therefore, abdominal wall ultrasonography and a complete abdominal ultrasound examination were requested for further characterization. Abdominal ultrasonography revealed hepatic volumetric redistribution (right lobe: 10.2 cm; left lobe: 7.5 cm), irregular liver contours, and diffusely heterogeneous parenchyma, without focal lesions or intrahepatic biliary duct dilation. The portal vein measured 1.2 cm in diameter, within the reported normal range (up to 1.3 cm). Paraumbilical collateral vessels were also identified, supporting the presence of portosystemic collateral circulation in the setting of suspected portal hypertension. The spleen measured 11.6 cm in longitudinal diameter, within normal limits, and no ascites was reported.

Doppler ultrasonography of the abdominal wall demonstrated dilation of the ascending portion of the left branch of the portal vein (Figure 2), raising suspicion of paraumbilical vein recanalization, which was subsequently confirmed during the same examination. Continuity was observed between the left portal vein branch and the ligamentum teres, with the vessel extending along the anterior abdominal wall toward the umbilicus. Extensive portosystemic collateral circulation was identified, including a recanalized paraumbilical vein and multiple superficial veins of the anterior abdominal wall, consistent with the imaging appearance of caput medusae (Figure 3). Color and spectral Doppler assessment demonstrated venous flow within these collateral vessels, with drainage into the inferior epigastric veins (Figures 4, 5).

**Figure 2:**
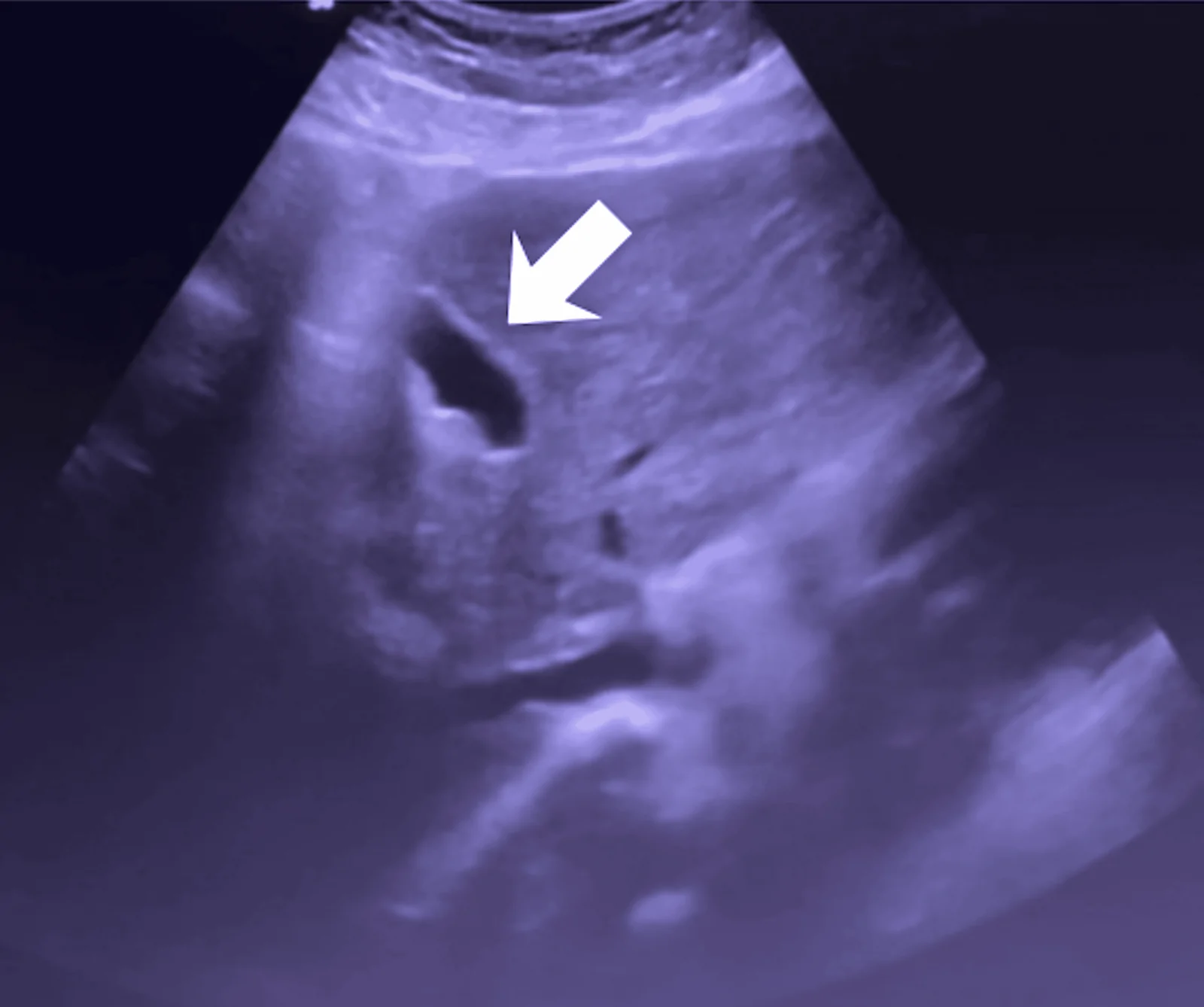
Significant dilatation of the ascending portion of the left portal vein branch.

**Figure 3:**
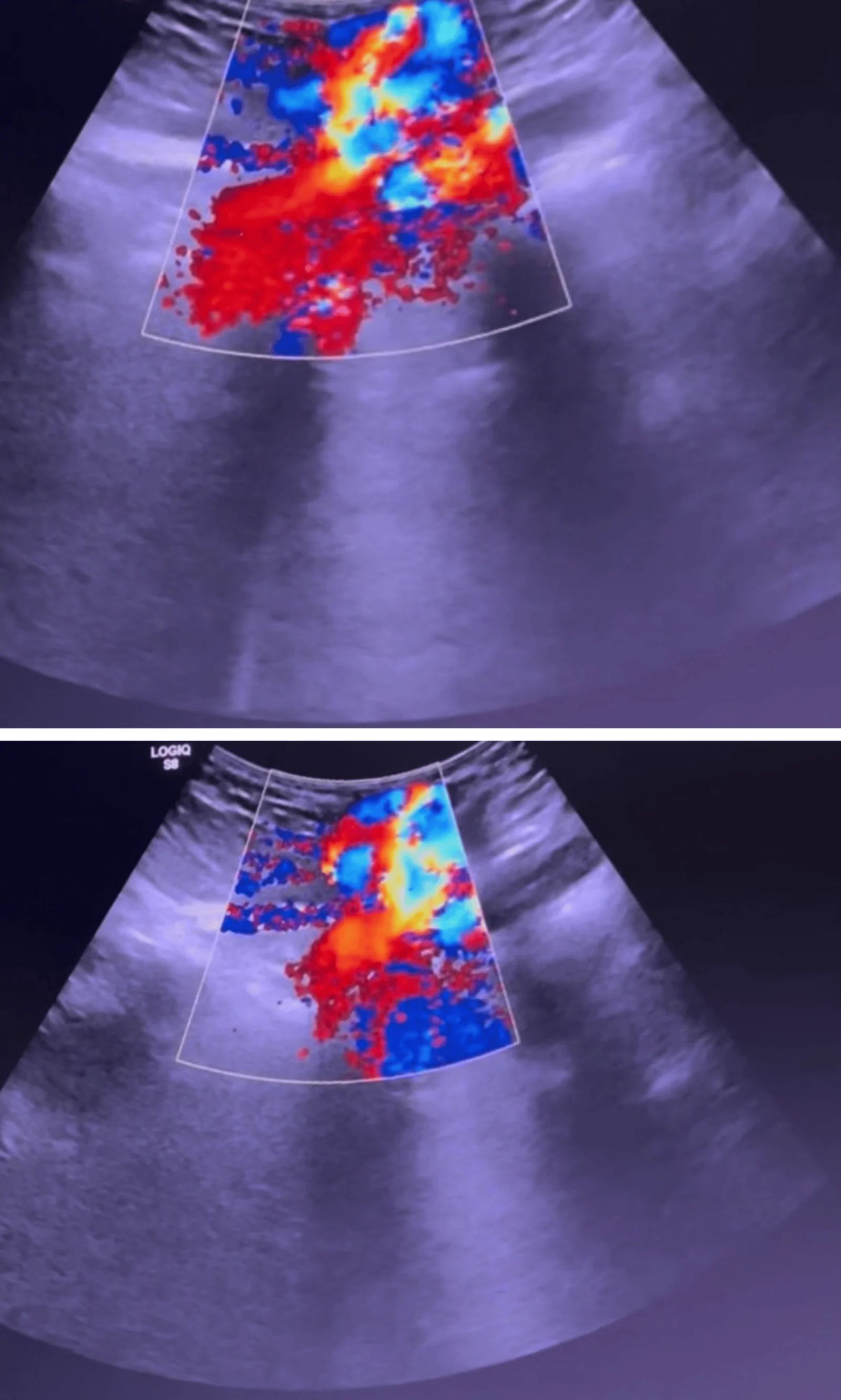
Prominent venous flow on Doppler ultrasonography in the paraumbilical region.

**Figure 4:**
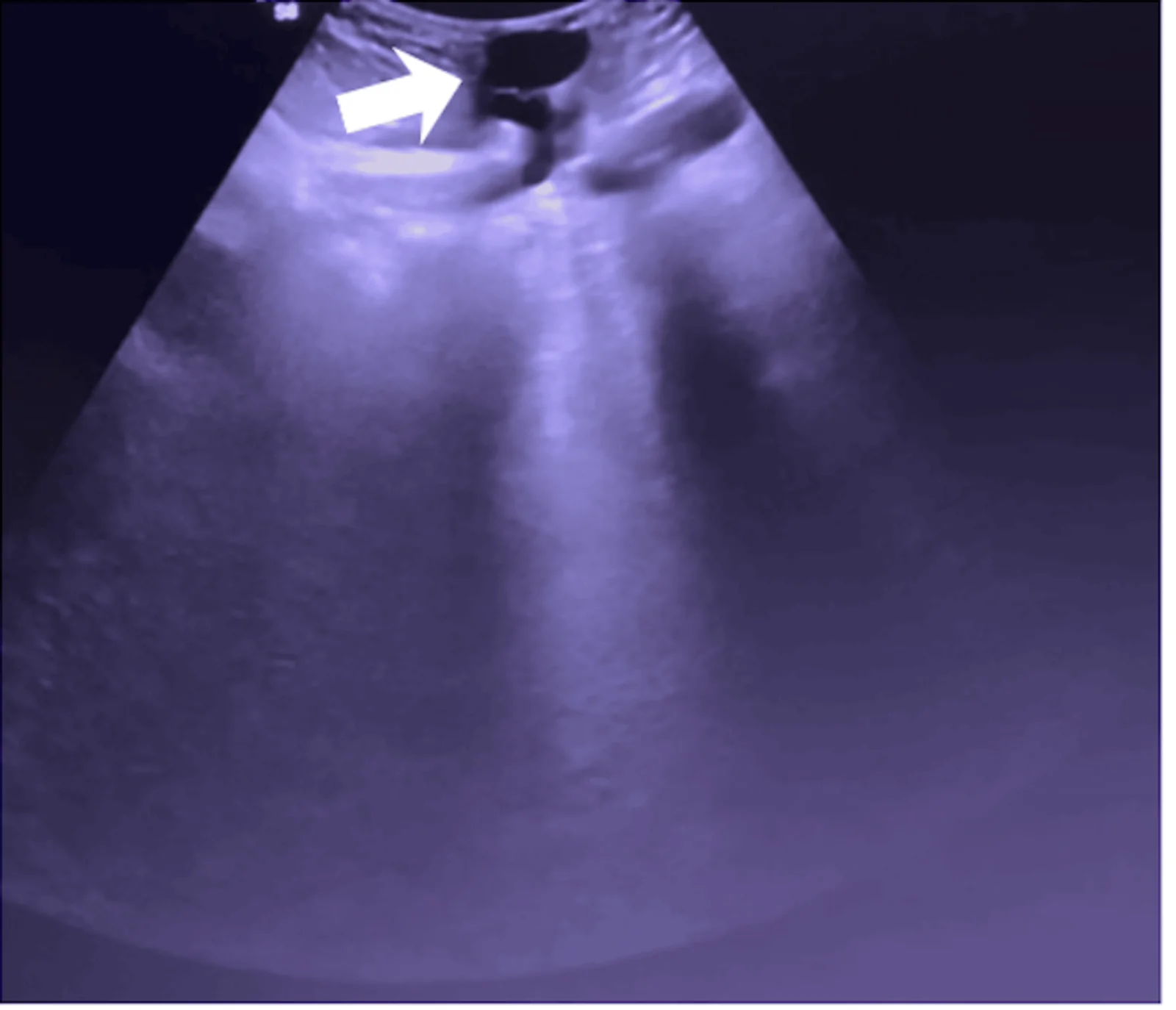
Dilatation of the paraumbilical vein at the subcutaneous level.

**Figure 5:**
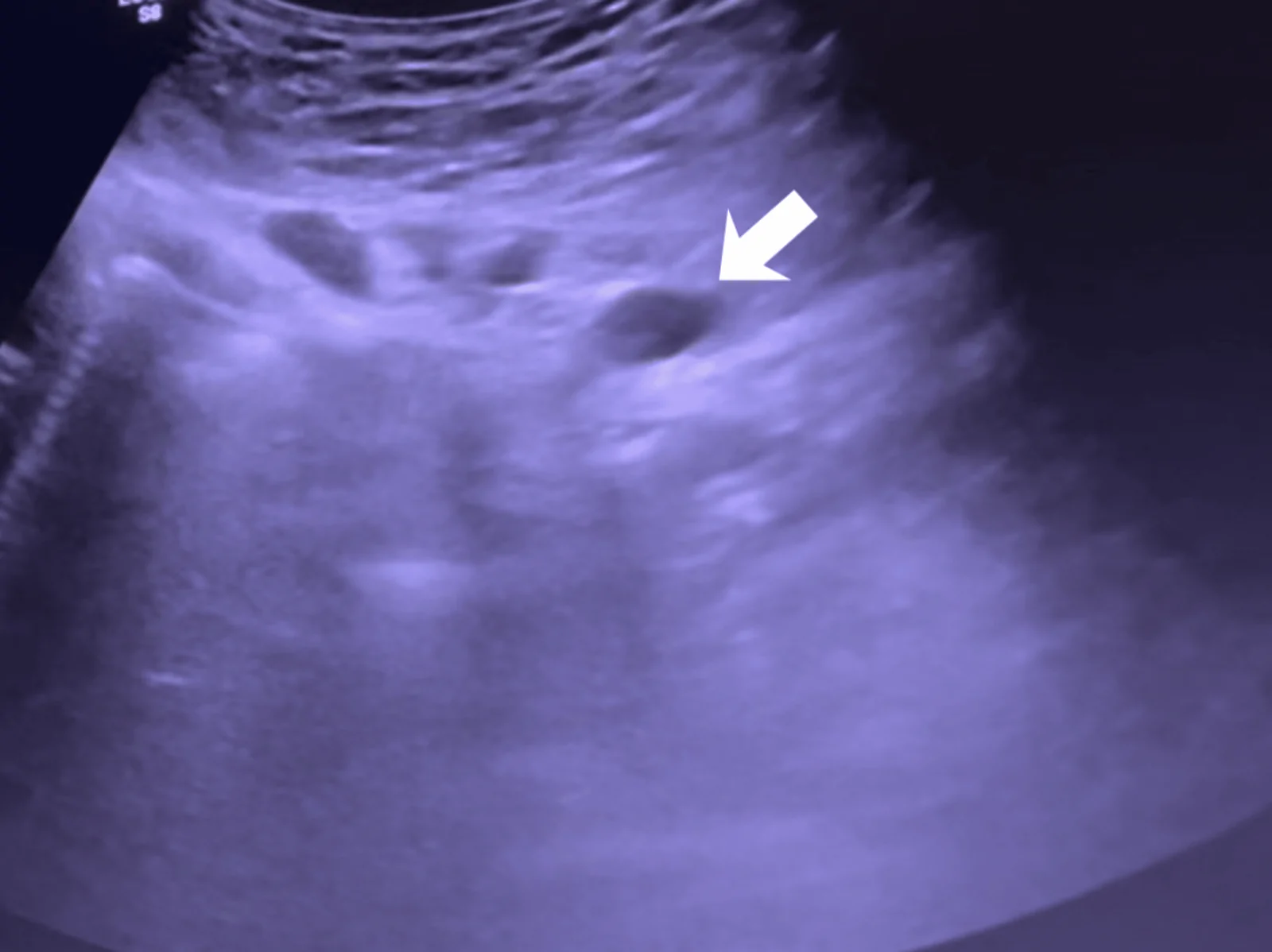
Inferior epigastric vein.

The identification of a recanalized paraumbilical vein and extensive superficial venous collaterals provided an explanation for the vascular component of the umbilical protrusion. The absence of an identifiable fascial defect or herniated abdominal contents favored a vascular etiology over an umbilical hernia. Although the imaging findings supported the presence of portosystemic collateral circulation associated with portal hypertension, portal pressure was not directly measured. Surgical intervention was contraindicated, and the patient continued under clinical follow-up in the Liver Institute of Pernambuco, Brazil. Further information regarding endoscopic screening for esophageal varices, subsequent management, and long-term outcomes was unavailable.

Table 2 summarizes the chronology of the patient's clinical course, diagnostic evaluation, and therapeutic management.

**Table 2:** Timeline of the clinical course, diagnostic evaluation, and management. The timeline summarizes the identification of thrombocytopenia and chronic hepatitis C virus infection, antiviral treatment and achievement of sustained virological response, subsequent detection of an umbilical protrusion, ultrasonographic and Doppler characterization of recanalized paraumbilical venous circulation, and the decision to contraindicate surgical intervention, with continued clinical follow-up. MELD: Model for End-Stage Liver Disease.

Timeline	Clinical events
February 2024	Initial investigation: Thrombocytopenia detected during routine preoperative testing for a urological procedure. Chronic hepatitis C virus (HCV) infection confirmed by reactive anti-HCV serology and detectable HCV-RNA.
March–May 2024	Antiviral treatment: Sofosbuvir/velpatasvir administered for 12 weeks, without adverse events or clinical complications.
August 2024	Virological outcome: Undetectable HCV-RNA, consistent with sustained virological response (SVR12). The patient remained asymptomatic during outpatient follow-up.
January 2026	Clinical presentation: Painless, compressible umbilical protrusion measuring approximately 2 cm, with normal overlying skin and no detectable venous hum. The patient expressed interest in elective surgical correction.
February 2026	Current evaluation: Persistent thrombocytopenia (92,000/µL), total bilirubin of 1.8 mg/dL, preserved albumin and renal function. Child–Pugh class A (5 points) and MELD 3.0 score of 11. No clinical evidence of hepatic decompensation.
February 2026	Abdominal ultrasonography: Irregular liver contours, heterogeneous parenchyma, hepatic volumetric redistribution, and paraumbilical collateral vessels. No ascites or splenomegaly.
February 2026	Abdominal wall Doppler ultrasonography: Recanalized paraumbilical vein connected to the left portal vein branch and ligamentum teres, with extensive superficial abdominal wall venous collaterals and Doppler-confirmed venous flow draining into the inferior epigastric veins.
February 2026, following imaging evaluation	Management and outcome: Surgical intervention was contraindicated following identification of the vascular abnormality. The patient continued clinical follow-up at the Liver Institute of Pernambuco, Brazil.

## Discussion

This case illustrates an important differential diagnosis of umbilical hernia in patients with chronic liver disease [2,3]. Caput medusae results from the development of portosystemic collateral circulation in response to chronic portal hypertension [2]. One of the main mechanisms underlying its formation is the recanalization of the paraumbilical vein, a remnant of the fetal circulation located within the falciform ligament, which originates from the umbilical portion of the left portal vein and normally obliterates after birth [4]. Once recanalized, this vein establishes communication between the portal venous system and the superficial veins of the anterior abdominal wall, producing the characteristic appearance of dilated veins radiating from the umbilicus [2,8]. Although caput medusae is considered a classic manifestation of portal hypertension, it has become less frequently encountered in contemporary clinical practice owing to earlier diagnosis and treatment of chronic liver disease and its complications [1,4].

Recanalization and dilatation of the paraumbilical vein in the setting of portal hypertension fall within the Cruveilhier-Baumgarten spectrum, which encompasses portosystemic collateralization around the umbilicus. The classical clinical manifestations may include caput medusae and a venous hum or murmur over the dilated vessels; however, these findings are not necessarily present in every patient. This distinction is relevant to the present case, in which imaging demonstrated a recanalized paraumbilical vein, although the characteristic superficial veins of caput medusae were not documented on physical examination or in the clinical photographs. Accordingly, the imaging finding is more appropriately discussed within the Cruveilhier-Baumgarten spectrum rather than considered definitive evidence of the clinical sign of caput medusae [2].

In the present case, the patient had been referred for surgical repair of a presumed umbilical hernia. However, clinical evaluation combined with imaging studies demonstrated that the umbilical protrusion actually corresponded to portosystemic collateral circulation secondary to portal hypertension. This finding underscores the importance of considering caput medusae in the differential diagnosis, as performing surgery without prior recognition of this condition may result in injury to recanalized venous collaterals, leading to severe and potentially fatal hemorrhage [2,3]. Therefore, imaging evaluation should precede any surgical intervention in patients with chronic liver disease presenting with a suspicious umbilical protrusion [4].

In this context, imaging modalities, particularly Doppler ultrasonography, play a pivotal role in the evaluation of these patients. In addition to identifying dilated paraumbilical vessels and recanalization of the paraumbilical vein, ultrasonography can demonstrate other imaging features suggestive of portal hypertension, including portal vein dilatation, portosystemic collateral vessels, splenomegaly, and ascites [4]. Doppler ultrasonography also enables assessment of the direction and velocity of portal venous blood flow, providing valuable information regarding the hemodynamic consequences of portal hypertension [4]. Although computed tomography and magnetic resonance imaging can also demonstrate anatomical changes associated with portal hypertension, these modalities have limited ability to estimate its severity and are primarily useful for detecting complications and delineating vascular anatomy [4]. More recently, liver elastography has improved the noninvasive assessment of chronic liver disease by estimating liver stiffness, thereby facilitating fibrosis staging and the identification of compensated advanced chronic liver disease. In contrast, conventional ultrasonography primarily evaluates liver morphology and signs of portal hypertension, although its sensitivity for detecting early or compensated cirrhosis is limited. These complementary modalities should be interpreted alongside clinical and laboratory findings to support diagnosis and risk stratification [4]. Thus, ultrasonography remains an accessible, noninvasive, and highly valuable tool for establishing the diagnosis, guiding clinical management, and preventing inappropriate surgical interventions [2,4]. Furthermore, the identification of a patent paraumbilical vein represents an important marker of portosystemic collateralization in portal hypertension and has been associated with greater hemodynamic impairment and more advanced liver disease, reinforcing the importance of recognizing this finding during imaging evaluation [8].

The main differential diagnoses include umbilical hernia, resulting from the protrusion of intra-abdominal contents through a defect in the abdominal wall; Sister Mary Joseph nodule, a periumbilical metastatic lesion most commonly associated with gastrointestinal or gynecological malignancies; isolated abdominal wall varices, which may occur in the absence of paraumbilical vein recanalization; venous malformations, congenital low-flow vascular anomalies that may present as compressible abdominal wall masses; primary tumors of the abdominal wall, such as liposarcoma; and urachal lesions, including infected urachal cysts, particularly when associated with pain, inflammatory signs, or an infraumbilical location [9]. Although dilated abdominal wall veins are classically associated with portal hypertension, they may also occur in the setting of inferior vena cava obstruction and, less commonly, superior vena cava obstruction [8].

As these entities often exhibit overlapping clinical and radiological features, the integration of clinical findings with imaging studies is essential for establishing the correct diagnosis and guiding appropriate therapeutic management [8].

Although portal hypertension is a common complication of liver cirrhosis [1,4], paraumbilical venous collaterals may present as an umbilical protrusion and pose a diagnostic challenge by mimicking an umbilical hernia [2]. This case highlights the importance of considering vascular causes, including findings within the Cruveilhier-Baumgarten spectrum, in the differential diagnosis of umbilical protrusions in patients with chronic liver disease. Appropriate imaging, including Doppler ultrasonography when a vascular etiology is suspected, may help characterize these lesions and inform management before surgical intervention [2,4].

## Conclusions

Caput medusae is a manifestation of portal hypertension that should be considered in the differential diagnosis of umbilical protrusions in patients with chronic liver disease. As abdominal wall vascular collaterals may mimic an umbilical hernia, careful clinical assessment and appropriate imaging, including Doppler ultrasonography when a vascular etiology is suspected, are important to characterize the lesion and inform management. This case highlights the importance of considering a vascular cause when evaluating umbilical protrusions in patients with cirrhosis, particularly before surgical intervention.

## Appendices

**Table 3:** Child-Pugh scoring system parameters. Adapted from Pugh et al. [6].

Parameter	1 point	2 points	3 points
Bilirubin	< 2 mg/dL	2–3 mg/dL	> 3 mg/dL
Albumin	> 3.5 g/dL	2.8–3.5 g/dL	< 2.8 g/dL
INR (international normalized ratio (prothrombin time))	< 1.7	1.7–2.3	> 2.3
Encephalopathy	Absent	Grade I or II	Grade III or IV
Ascites	Absent	Easily controlled	Refractory

**Table 4:** Child-Pugh scoring system results. Adapted from Pugh et al. [6].

Total score	Child-Pugh class
5–6 points	Class A
7–9 points	Class B
10–15 points	Class C

Formula for calculating the MELD 3.0 score, adapted from Kim et al. [7]:

${MELD\ 3.0} = 4.56 \times \ln(\mathrm{bilirubin}) + 0.82 \times (137 - \mathrm{Na}) - 0.24 \times (137 - \mathrm{Na}) \times \ln(\mathrm{bilirubin}) + 9.09 \times \ln(\mathrm{INR}) + 11.14 \times \ln(\mathrm{creatinine}) + 1.85 \times (3.5 - \mathrm{albumin}) - 1.83 \times (3.5 - \mathrm{albumin}) \times \ln(\mathrm{creatinine}) + 6$

Abbreviations: MELD, Model for End-Stage Liver Disease; INR, international normalized ratio; Na, serum sodium; ln, natural logarithm.
